# Genome wide comparative analysis of the effects of PRMT5 and PRMT4/CARM1 arginine methyltransferases on the *Arabidopsis thaliana* transcriptome

**DOI:** 10.1186/s12864-015-1399-2

**Published:** 2015-03-17

**Authors:** Carlos E Hernando, Sabrina E Sanchez, Estefanía Mancini, Marcelo J Yanovsky

**Affiliations:** Fundación Instituto Leloir, Instituto de Investigaciones Bioquímicas de Buenos Aires–Consejo Nacional de Investigaciones Científicas y Técnicas de Argentina, Buenos Aires, Argentina; Molecular and Computational Biology Section, University of Southern California, Los Angeles, CA 90089 USA

## Abstract

**Background:**

Methylation at arginine residues (R) is an important post-translational modification that regulates a myriad of essential cellular processes in eukaryotes, such as transcriptional regulation, RNA processing, signal transduction and DNA repair. Arginine methylation is catalyzed by a family of enzymes known as protein arginine methyltransferases (PRMTs). PRMTs are classified as Type I or Type II, depending on the position of the methyl group on the guanidine of the methylated arginine. Previous reports have linked symmetric R methylation to transcriptional repression, while asymmetric R methylation is generally associated with transcriptional activation. However, global studies supporting this conclusion are not available.

**Results:**

Here we compared side by side the physiological and molecular roles of the best characterized plant PRMTs, the Type II PRMT5 and the Type I PRMT4, also known as CARM1 in mammals. We found that *prmt5* and *prmt4a;4b* mutants showed similar alterations in flowering time, photomorphogenic responses and salt stress tolerance, while only *prmt5* mutants exhibited alterations in circadian rhythms. An RNA-seq analysis revealed that expression and splicing of many differentially regulated genes was similarly enhanced or repressed by PRMT5 and PRMT4s. Furthermore, PRMT5 and PRMT4s co-regulated the expression and splicing of key regulatory genes associated with transcription, RNA processing, responses to light, flowering, and abiotic stress tolerance, being candidates to mediate the physiological alterations observed in the mutants.

**Conclusions:**

Our global analysis indicates that two of the most important Type I and Type II arginine methyltransferases, PRTM4 and PRMT5, have mostly overlapping as well as specific, but not opposite, roles in the global regulation of gene expression in plants.

**Electronic supplementary material:**

The online version of this article (doi:10.1186/s12864-015-1399-2) contains supplementary material, which is available to authorized users.

## Background

Post-translational modification of proteins is a typical mark of signal transduction pathways through which organisms are able to react quickly to changes in their surrounding by expanding the structural and functional diversity of the proteome. Arginine methylation is a common post- translational modification in eukaryotic organisms, and is catalyzed by a family of enzymes known as Protein Arginine Methyltransferases (PRMTs). This post-translational modification modulates a myriad of cellular processes through its effects on proteins involved in the regulation of chromatin structure, transcription, RNA processing, signal transduction and cellular differentiation, among other processes [1-6]. PRMTs are classified in four groups: type I PRMTs that generate ω-N^*G*^-monomethyl arginine (MMA) and ω-N^*G*^,N^*G*^-asymmetric dimethylarginines (aDMA), type II PRMTs that generate ω-N^*G*^-monomethyl arginine and ω-N^*G*^,N^*G*^-symmetric dimethylarginines (sDMA), type III PRMTs that generate only ω-N^*G*^-monomethyl arginine, and type IV that generate only δ-N^*G*^-monomethyl arginine [7]. Currently, four different genes encoding PRMTs have been described in *Saccharomyces cerevisiae*, eight in *Oryza sativa*, and nine in humans, *Drosophila* and *Arabidopsis thaliana* [8,9]. In mammals, there are six well characterized arginine methyltransferases, five of them corresponding to type I PRMTs (PRMT1, PRMT3, PRMT4/CARM, PRMT6 and PRMT8); while the only type II PRMT known so far is PRMT5. It has been proposed that there is no major redundancy between these enzymes, at least in mammals, since knock-out mice for each PRMT display clearly different phenotypes [2].

It is known that proteins that possess glycine and arginine- rich (GAR) motifs are often targets of PRMTs. Transcriptional regulation by methylation of histones and non-histone proteins is one of the more characterized functions of PRMTs. It has been reported that PRMT1 methylates Arg 3 of histone H4, PRMT4 methylates Arg 2, Arg 17 and Arg 26 of histone H3 and that PRMT5 methylates Arg 8 of histone H3 and Arg 3 of histone H4. In mammals, formation of aDMA in histones by PRMT1 and PRMT4 participates in gene activation while formation of sDMA by PRMT5 is associated with gene repression [3,10]. Arginine methylation has also been associated with the regulation of the initiation and elongation steps of transcription. The recruitment of PRMT4 to transcriptional promoters results in methylation of histone acetyltransferases resulting in a positive effect on transcription [11], while PRMT5 methylates the transcriptional elongation factor SPT5 regulating its interaction with RNA polymerase II [12]. Another well characterized role of PRMTs is the regulation of RNA processing. RNA binding proteins (RBPs) fulfill numerous tasks ensuring the proper processing and folding as well as the stabilization and localization of RNAs and mRNA translation. These proteins represent major targets for PRMTs because most hnRNPs possess GAR motifs. PRMTs are also known to methylate Sm spliceosomal proteins B, B’, D1 and D3, and this mediates their assembly into mature small nuclear ribonucleoprotein particles (snRNPs), and has been associated with a role for PRMT5 in the regulation of pre-mRNA splicing [13-15]. On the other hand, PRMT4 regulates the coupling of transcription and mRNA processing through methylation of splicing factors [16-18].

Strikingly, only a few studies have compared side by side the roles of distinct PRMTs in the regulation of different physiological and molecular processes. In fact, several of these studies showed that PRMT4 and PRMT5 simultaneously control myogenesis, with both proteins having similar positive roles in the control of expression of genes known to play a key role regulating this developmental process [19-23]. This is at odds with the current view that assumes that PRMT4, a type I PRMT, acts as a co-activator of gene expression, while PRMT5, the main type II PRMT, acts as a transcriptional repressor [3,4,10,24]. Whether the similar positive role that PRMT5 and PRMT4 play in the control of myogenesis is the exception or the rule is not known. Discriminating between these two alternatives requires a side-by-side comparison of the effects of PRMT5 and PRMT4 on gene expression at a genome-wide level.

Interestingly, both PRMT5 and PRMT4 have been proposed to play key roles in the regulation of pre-mRNA splicing. As previously reviewed, this regulatory role may be exercised through direct methylation of core spliceosomal proteins or, alternatively or in addition, through regulation of the coupling between transcription and mRNA processing [1,17,18,25-27]. However, while genome-wide analyses of pre-mRNA splicing have been conducted to characterize *prmt5* deficient plants or animals, similar global analysis are missing for mutants affected in *PRMT4*.

Plants are ideal organisms to conduct a side-by-side comparison of the roles of PRMT5 and PRMT4 on gene expression and RNA processing, because mutant plants lacking these proteins are viable and fertile, while defects in these two genes are lethal in mammals. In *Arabidopsis thaliana* there are seven type I arginine methyltransferases (PRMT1a, PRMT1b, PRMT3, PRMT4a, PRMT4b, PRMT6 and PRMT10) and, as in mammals, one type II enzyme (PRMT5). To date, the most intensively studied PRMT in plants is PRMT5, which controls flowering time, circadian rhythms photomorphogenic development and salt tolerance acting on epigenetic regulation of gene expression and on pre-mRNA splicing of a sub-set of genes [25,26,28-32]. Regarding type I PRMTs, it has been shown that PRMT10, PRMT1b, and PRMT4a together with PRMT4b, are all involved in the regulation of flowering time [33-35]. Both type I and type II PRMTs appear to regulate flowering time in *Arabidopsis thaliana* through effects on *FLC* expression. FLC is a MADS box transcription factor that represses flowering, and *prmt5*, *prmt10* mutants and the *prmt4a;4b* double mutant all exhibit increased *FLC* expression, which is partially responsible for the delayed flowering observed in these mutant plants [28,33,35].

So far, the only phenotype that has been reported for *prmt4a;4b* mutant plants is delayed flowering [35]. Interestingly, this mutant resembles *prmt5* plants at the morphological level, showing some degree of growth retardation and dark green leaves. However, whether *prmt4a;4b* also exhibits other physiological alterations present in *prmt5* mutants such as defects in circadian clock function, photomorphogenic development or salt stress tolerance is not known.

In this study we compared side by side the role of PRMT5 and PRMT4s in the regulation of several physiological processes in *Arabidopsis thaliana*, and coupled this with a genome-wide comparison of their effects on gene expression and pre-mRNA splicing using RNA-seq. We found that *prmt5* and *prmt4a;4b* mutants not only displayed similar alterations in flowering time regulation, but also exhibited reduced inhibition of hypocotyl elongation under both red and blue light, revealing defects in light signaling. In addition, similarly to what has been reported for *prmt5* mutants, we found that *prmt4a;4b* double mutants exhibited reduced tolerance to salt stress. However, in contrast to what is observed in *prmt5* mutants, the *prmt4a;prmt4b* double mutant did not exhibit alterations in circadian rhythms. RNA-seq data showed that both mutants display similar alterations in the expression of genes related to: transcription, mRNA processing, mRNA splicing, translation, light signaling, response to hormones and both abiotic and biotic stress. In addition, we also found that these mutants have similar alterations in alternative splicing (AS) of genes related to translation, light signaling, response to hormones and both abiotic and biotic stress. Finally, we observed a significant number of novel intron retention events in both mutants, revealing alterations in a subset of constitutive as well as AS events in those plants.

Hence, this study shows that in *Arabidopsis thaliana*, type I PRMT4s and type II PRMT5 regulate overlapping as well as distinct physiological processes, most likely through similar effects on gene expression and pre-mRNA splicing of a subset of key regulatory genes. Additionally, this study is the first to report an analysis of the role of PRMT4s on pre-mRNA splicing at a global level.

## Results and discussion

### PRMT5 and PRMT4 control overlapping as well as distinct physiological processes

**Figure 1:**
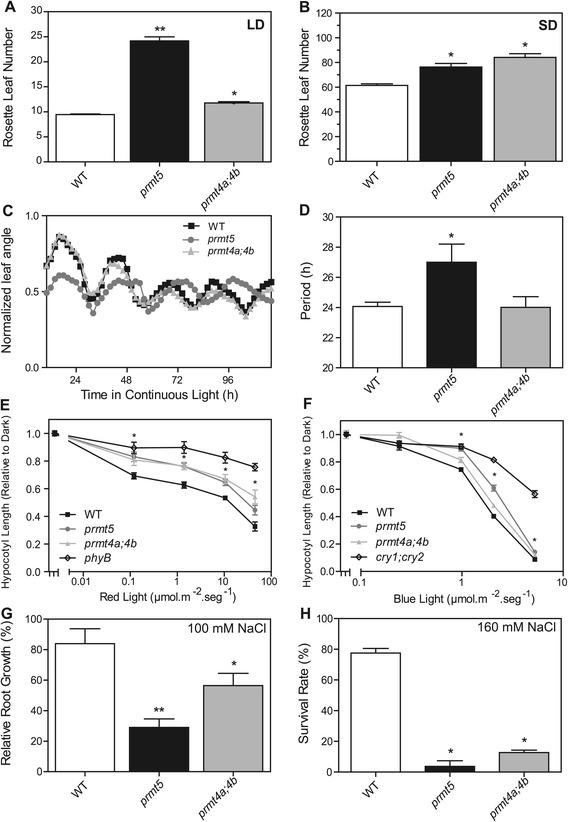
**Side by side comparison of the physiological roles of PRMT5 and PRMT4. (A and B)** Flowering time measured as the number of rosette leaves at bolting in Long Day **(A)** and Short Day **(B)** photoperiods. ANOVA followed by a Tukey’s multiple comparision test was used to evaluate the statistical significance of the differences observed between genotypes. Errors bars indicate SD (**: significantly different than WT and *prmt4a;4b*, *: significantly different than WT, both cases p ≤ 0,05). **(C)** Circadian rhythms of leaf movement in continuous light (N = 10). **(D)** Period of the circadian rhythms was estimated with BRASS 3.0 software (*: significantly different, p ≤ 0,05). **(E and F)** Hypocotyl length of seedlings grown under continuous red light **(E)** or continuous blue light **(F)** (N = 6 replicates of 10 seedlings each, *: significantly different than WT, p ≤ 0,05). **(G)** Comparison of root growth on MS medium with 100 mM NaCl. Root growth was measured relative to controls. More than 30 roots were measured for each data point. Data represents the mean with SD of three independent experiments (**: significantly different than WT and *prmt4a;4b*, *: significantly different than WT, both cases p ≤ 0,05). **(H)** Salt tolerance was assessed through the analysis of survival rate on MS medium containing 160 mM NaCl. Data represents the mean with SD of three independent experiments (N > 50, *: significantly different than WT, p ≤ 0,05).

Before conducting a genome-wide comparison of the roles of PRMT5 and PRMT4 in the regulation of gene expression and RNA processing in *Arabidopsis thaliana*, we performed a side by side analysis of the effect of these genes on the control of several developmental and physiological processes. In particular, we focused the analysis on clock-associated processes, such as flowering time regulation, photomorphogenic responses and circadian rhythms, as well as on salt stress tolerance. All these physiological and developmental processes have previously been shown to be affected in *prmt5* mutants but have not been characterized in *prmt4a;4b* mutants, with the exception of flowering time. Indeed, as previously reported individually for *prmt5* and *prmt4a;4b*, [28,35], both mutants showed a late flowering phenotype compared to wild-type plants of the Col-0 accession. However, while both mutants displayed a clear and similar late flowering phenotype under short day photoperiods, the late flowering phenotype of *prmt5* was much stronger than that of *prmt4a;4b* mutant plants under long day photoperiods (Figure 1A and B).

We and others have previously shown that PRMT5 plays a key role in the regulation of circadian rhythms in Arabidopsis [25,32], but whether PRMT4 contributes to the regulation of clock function is not known. To evaluate this we monitored circadian rhythms in leaf movements in wild-type plants, *prmt5* and *prmt4a;4b* mutant plants. Interestingly, while *prmt5* mutant plants showed the previously described long period phenotype for leaf movement, the rhythms observed in *prmt4a;4b* mutants were similar to those of wild-type plants (Figure 1C and D). In order to analyze the role of these PRMTs in photomorphogenesis we evaluated light inhibition of hypocotyl elongation during de-etiolation in seedlings exposed to different fluence rates of red and blue light. We found that both *prmt5* and *prmt4a;4b* mutants were hyposensitive to red light at all fluence rates tested (Figure 1E). A similar phenotype was observed for both mutants under blue light as well (Figure 1F).

PRMT5 has been shown to regulate salt stress tolerance in Arabidopsis [30]. To test if PRMT4a, together with PRMT4b, were also involved in the regulation of this physiological response, we analyzed root growth, a process affected by high salt concentrations. Root length of wild-type plants grown on MS medium containing 100 mM NaCl was approximately 80% of that shown by wild-type plants grown on MS medium alone. In contrast, root length of *prmt5* and *prmt4a;4b* mutants grown on medium containing the same concentration of salt was only 30% and 50%, respectively, relative to that of plants from these genotypes grown on MS medium (Figure 1G). We also conducted a survival rate assay and found that growth of both *prmt5* and *prmt4a;4b* mutants was almost completely inhibited in medium containing 160 mM NaCl, while wild-type plants displayed only a slightly inhibited growth rate under this condition (Figure 1H). Altogether this shows that PRMT5 and, to a lesser extent PRMT4a, together with PRMT4b, are all involved in the control of salt stress tolerance in Arabidopsis.

### Impact of PRMT5 and PRMT4s on genome wide gene expression

**Figure 2:**
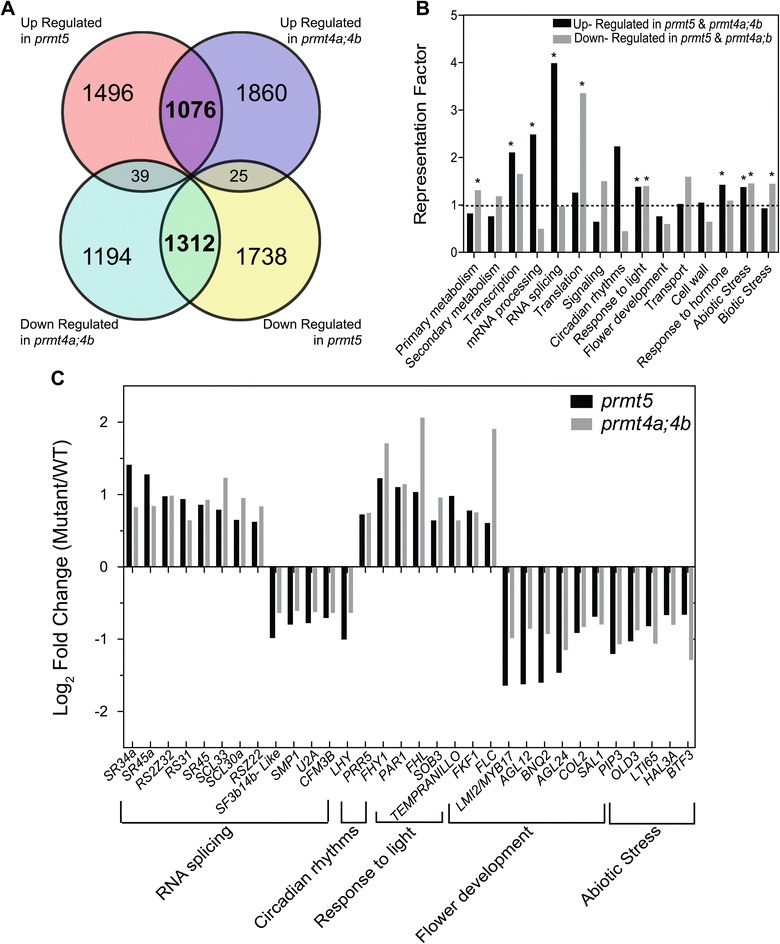
**Impact of PRMT5 and PRMT4 on genome wide gene expression. (A)** Overlap between differentially expressed genes in both *prmt5* and *prmt4a;4b* mutants (Log_2_ FC >|0,58| and FDR ≤ 0,1). **(B)** Representation Factor of genes co- regulated by PRMT5 and PRMT4s in the selected functional categories. *: indicates over-representation compared to random. Significance was assessed using a hypergeometric test (p ≤ 0,05). **(C)** Log_2_ Fold Change of representative genes co- regulated by PRMT5 and PRMT4s (FDR ≤ 0,1).

In order to study the extent of the regulatory impact of PRMT5 and PRMT4s on gene expression we analyzed the transcriptome of wild-type, *prmt5,* and *prmt4a;b* plants grown under standard non-stressful conditions (continuous white light at 22°C) using RNA-seq. We found 2604 genes over-expressed and 3075 under-expressed in *prmt5* mutants, as well as 2959 genes over-expressed and 2545 under-expressed in *prmt4a;4b* mutants, relative to wild-type plants. Strikingly, many of the differentially expressed genes were similarly affected in both mutants, with 1076 and 1312 genes over-expressed or under-expressed in common, respectively. On the other hand, only 64 genes, out of a total of 5679 and 5504 genes differentially expressed in either *prmt5* or *prmt4a;b,* respectively, were antagonistically affected in the mutants. Therefore, it is highly unlikely that the PRMTs analyzed here exhibit opposite biological and molecular roles (Figure 2A).

The differentially expressed genes that were similarly affected in both mutants were categorized into functional groups based on Gene Ontology. Fifteen functional categories of our interest were examined in detail determining the representation factor for each category. The representation factor is the number of overlapping genes observed divided by the expected number of overlapping genes drawn randomly from two independent groups. Among the up-regulated genes, we found a significant enrichment (Representation Factor > 1; p-value <0.05) for categories corresponding to transcription, mRNA processing, RNA splicing, response to light, response to hormones, abiotic stress and biotic stress. For down-regulated genes, we found significant enrichment for genes associated with primary metabolism, translation, response to light, abiotic stress and biotic stress (Figure 2B). None of the categories studied here displayed a statistically significant under-representation (Representation Factor < 1; p-value <0.05). Finally, we also found a number of genes that were significantly affected only in *prmt5* or *prmt4a;b* mutants, some of which may be responsible for the partially distinct phenotypes of the mutants, such as the differential effect on circadian rhythmicity (Additional file 1). Altogether, our physiological and molecular analysis suggests that these PRMTs regulate most of the mentioned processes controlling the expression of a common set of genes. Indeed, when we analyzed in detail the identity of the co-regulated genes associated with the biological processes mentioned above, we found that many of them corresponded to transcription factors with key regulatory roles in the associated processes or pathways (e.g. *PRR5*, *PAR1*, and *FLC* among others). Thus, this suggests that the PRMTs studied here may regulate a common set of biological processes regulating directly or indirectly a few key major regulatory genes, which then control the expression of hundreds of genes associated with different biological responses (Figure 2C). Our data resembles the results shown for muscle differentiation in mouse and zebrafish, where PRMT5 and PRMT4 positively regulate the expression of genes involved in myogenesis [21-23]. Therefore, the results of our genome-wide analysis do not support the idea that PRMT5 and PRMT4 act specifically as transcriptional repressor and activator, respectively, as previously suggested based on the analysis of a few genes. In fact, there is an increasing number of publications revealing roles for PRMT5 as a transcriptional activator and roles for PRMT4 as a transcriptional repressor [11,36-39]. It is worth mentioning that we cannot determine if the changes in mRNA levels observed between wild-type and *prmt* mutant plants are mediated by alterations in histone methylation, or by changes in the methylation status of non-histone targets that regulate gene expression at the transcriptional and/or post-transcriptional levels.

### Impact of PRMT5 and PRMT4s on genome-wide AS

**Figure 3:**
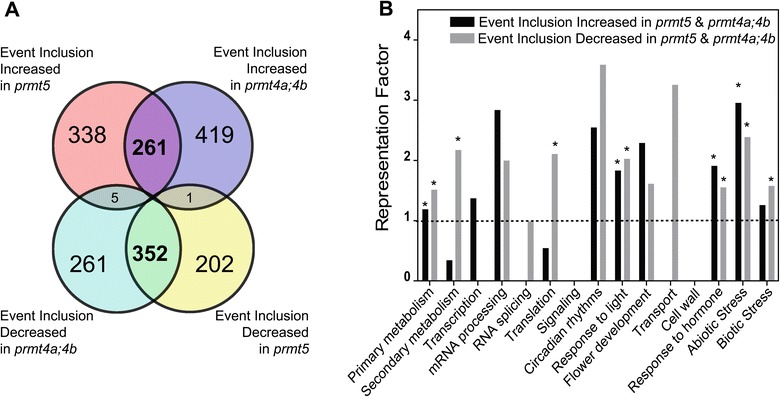
**Impact of PRMT5 and PRMT4s on genome wide AS. (A)** Overlap between significantly altered alternative splicing events. **(B)** Representation Factor of alternative splicing events co- regulated by PRMT5 and PRMT4s in the selected functional categories. *: indicates over-representation compared to random. Representation factor significance was assessed using a hypergeometric test (p ≤ 0,05).

**Figure 4:**
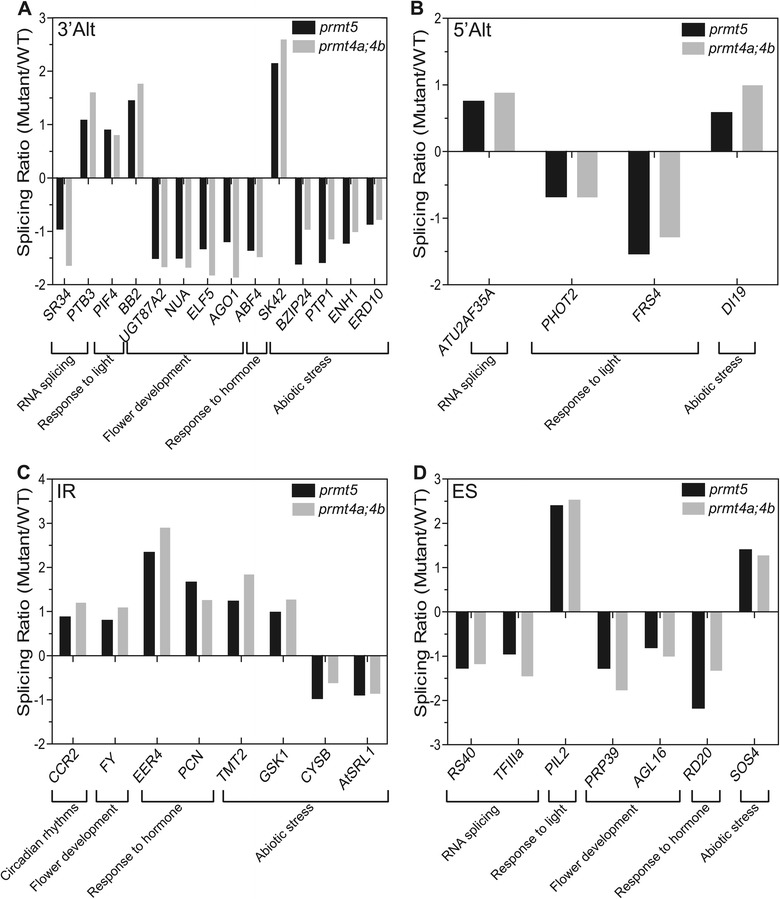
**Alternative Splicing Events altered in** ***prmt5*** **and** ***prmt4a;4b*** **. (A)** Splicing Ratio of 3′ alternative splicing events affected in both mutants. **(B)** Splicing Ratio of 5′ alternative splicing events altered in both mutants. **(C)** Splicing Ratio of Intron Retention events affected in both mutants. **(D)** Splicing Ratio of Exon Skipping events altered in both mutants. The GO category to which each gene belongs is indicated at the bottom of each panel.

Several reports have previously suggested a key role for type I and type II arginine methyltransferases in the regulation of pre-mRNA splicing. Indeed, the role of PRMT5 in this process has been well supported by genome-wide analyses of pre-mRNA splicing in *prmt5* mutant plants as well as in mammalian cells with reduced *PRMT5* expression. In contrast, our knowledge of the role of PRMT4 on pre-mRNA splicing is still limited to its effect on a few individual splicing events [17,18,26,29,32]. To characterize and compare the roles of *Arabidopsis* PRMT5 and PRMT4 on pre-mRNA splicing, we evaluated their effects on annotated AS events from genes expressed above a minimal threshold level in all genotypes. We found significant alterations in 1137 AS events in *prmt5* mutants and in 1290 in *prmt4a;4b* mutants, representing 19 and 21% of all AS events evaluated, respectively. Among the altered AS events identified, 261 exhibited increased inclusion and 352 decreased inclusion simultaneously in both mutants (Figure 3A). No significant differences were observed in the distribution of the AS categories 5′ and 3′ alternative splicing site, intron retention and exon skipping (Additional file 2). The AS events affected in common in both mutants were classified into functional categories, as described for the expression analysis, and evaluated for enrichment of specific categories in this data-set relative to their frequency in the genome. We found a significant over-representation for categories such as primary metabolism, response to light, response to hormones and abiotic stress (Figure 3B). In addition, we found alterations in specific genes associated with salt stress, RNA processing/splicing and flowering time regulation (e.g. *ATU2AF35A*, *ELF5*, *SOS4*), which could be at least partially responsible for the mutant phenotypes (Figure 4A, B, C and D). It is worth mentioning that some of the AS changes observed were specific for each mutant. In particular, the alteration in AS previously reported for the clock gene *PRR9* (*At2g46790*) [25] was clearly observed in *prmt5* but not in *prmt4a;b* mutants (Additional file 3). Indeed, a change in AS of *PRR9* is thought to be responsible for the circadian defect present in *prmt5* mutants [25]. Lack of effect of PRMT4s on this AS event is therefore consistent with the absence of a circadian phenotype in this mutant. Taken together, both mutants analyzed displayed significant alterations in the regulation of annotated AS events highlighting a key role for both PRMT5 and PRMT4 in the regulation of AS. In contrast to what we observed for gene expression, we did not find an over-representation of genes related to RNA processing among those affected at the AS level in the *prmt5* and *prmt4a;4b* mutants, suggesting that alterations in this process most likely result from effects on the expression of genes encoding splicing factors as well as from the post-translational regulation of specific proteins involved in RNA processing. On the other hand, processes such as primary metabolism, translation, response to light, hormone responses, and abiotic stress tolerance, all seem to be affected through changes in both gene expression and AS.

### Analysis of the effects of PRMT5 and PRMT4s on constitutive pre-mRNA splicing

To evaluate the role of PRMT5 and PRMT4 on constitutive splicing, we characterized the impact of mutations in these genes on splicing of all introns not annotated as alternatively spliced, which are present in genes expressed above a threshold level in all genotypes (Additional file 4). We found 2506 introns with increased retention in *prmt5* and 1143 in *prmt4a;4b* mutants relative to wild-type plants. This represents 3.1 and 1.4% of all introns studied in *prmt5* and *prmt4a;4b* mutants, respectively. Interestingly, a much larger effect was observed in introns annotated as alternatively spliced, with 17.7% of these affected in *prmt5* mutants and 17.8% in *prmt4a;4b* mutants. Thus, these results clearly indicate that PRMT5 and PRMT4s have much larger impact on alternative compared to constitutive splicing, as was previously reported for *prmt5* in *Arabidopsis* based on data from tiling arrays and a HR RT-PCR panel of well characterized AS events, in contrast to what was observed for mammals [25,27].

**Figure 5:**
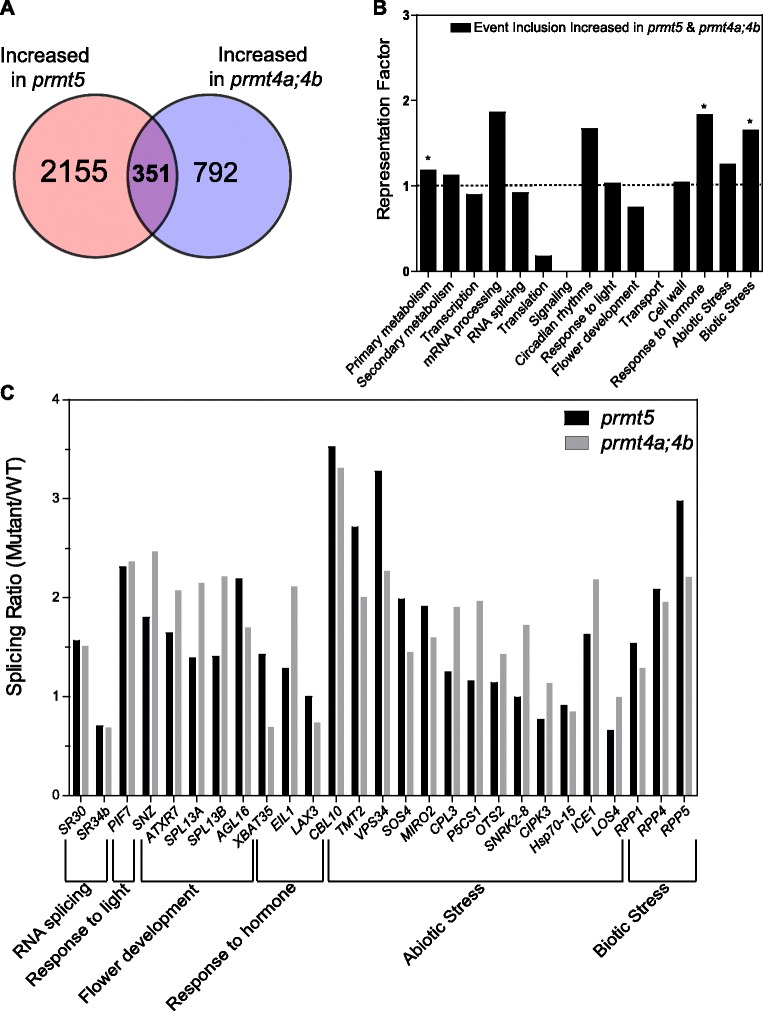
**Analysis of the effects of PRMT5 and PRMT4s on constitutive pre-mRNA splicing. (A)** Overlap between introns with increased retention in both mutants. **(B)** Representation Factor of introns whose retention increased simultaneously in *prmt5* and *prmt4a;4b* in the selected functional categories. *: indicates over-representation compared to random, representation factor significance was assessed using a hypergeometric test (p ≤ 0,05). **(C)** Splicing Ratios of some representative events showing increased inclusion in both mutants is displayed (FDR ≤0,1). The GO category to which each gene belongs is indicated at the bottom of each panel.

Interestingly, many of the increased intron retention events identified were similarly affected in *prmt5* and *prmt4a;4b* mutants (Figure 5A), as shown for gene expression and AS. We then categorized these common set of intron retention events using Gene Ontology, and found a significant enrichment in the categories corresponding to primary metabolism, response to hormones and biotic stress (Figure 5B). We also found several intron retention events associated with genes involved in the regulation of RNA splicing, light signaling, flowering, hormone signaling, and abiotic/biotic stress (Figure 5C). This data set supports the idea that PRMT5 and PRMT4 regulate the aforementioned processes, at least in part, through their effects on the regulation of pre-mRNA splicing of genes associated with them.

**Figure 6:**
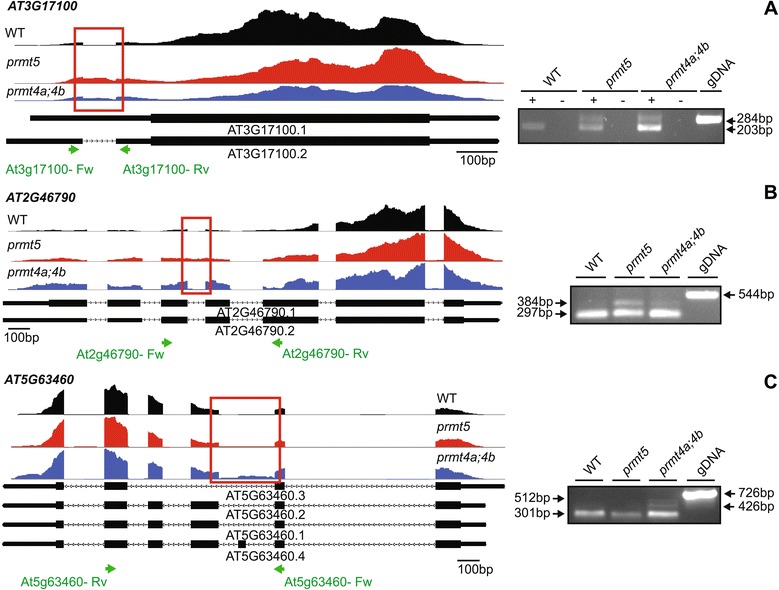
**RT-PCR Analysis of alternative splicing events.** RT-PCR validation of three events identified through RNA-seq. **(A)** Event affected in both mutants simultaneously. **(B)** Event affected only in *prmt5*. **(C)** Event affected only in *prmt4a;4b*. The read density map for each event evaluated is displayed (WT: black, *prmt5*: red, *prmt4a;4b*: blue). A scheme describing each gene is displayed below the read density maps, with exons and introns displayed as boxes and lines respectively. A red square encloses the measured event, while green arrows display the position of the oligo used for the RT-PCR measurement. An image of the agarose gel with the RT- PCR amplicons is displayed next to the read density maps. Black arrows indicate amplicon sizes. +: retrotranscriptase added, −: retrotranscriptase not added, gDNA: genomic DNA control. Each image represents one of three biological replicates measured.

Then, we validated alterations in pre-mRNA splicing detected using RNA-seq for three splicing events. One of this was an intron retention event associated with the gene *AT3G17100*, which was similarly affected in both *prmt5* as well as *prmt4a;4b* mutants (Figure 6A). Another event validated was an intron retention event associated with the core clock gene *AT2G46790*, also known as *PRR9*, which increased in *prmt5* and was not affected in *prmt4a;4b* (Figure 6B)*.* Finally, we assessed a multiple splicing event that was affected only in *prmt4a;4b* at the gene *AT5G63460*, which exhibited an increased retention of the intron 2 reported as constitutively spliced and, simultaneously, displayed an increased retention of the exon 3 that is reported as an alternative exon skipping event (Figure 6C).

**Figure 7:**
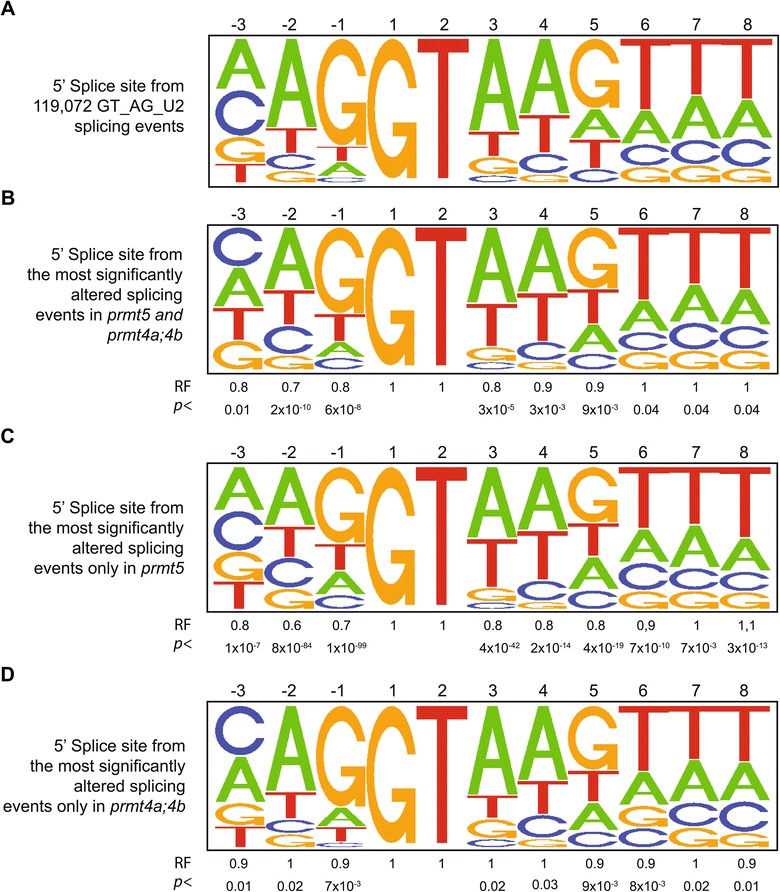
**Bioinformatic analysis of donor splice-site sequences.** Pictograms showing the frequency distribution of nucleotides at the 5′ splice site of **(A)** 119,072 GT_AG_U2 *Arabidopsis* introns, **(B)** the most significantly intron retention events whose splicing were altered simultaneously in both *prmt5* and *prmt4a;4b*, **(C)** the most significantly intron retention events altered only in *prmt5* and **(D)** the most significantly intron retention events altered only in *prmt4a;4b*. The representation factor (RF) is the frequency in the data set of interest divided by the total frequency. For each RF a p-value was calculated using the hypergeometric test.

Finally, we evaluated whether there was any change in the splice-site sequences of the intron retention events affected in *prmt* mutants compared to the consensus splice-site sequence of all introns present in the *Arabidopsis* genome (Figure 7A)*.* Interestingly, as previously reported for *prmt5* mutants [25], we found that the donor splice site sequences of the splicing events affected only in *prmt5* mutants, or simultaneously altered in *prmt5* and *prmt4a;b* mutants, displayed an under-representation of the consensus A and G nucleotides present in the −2 and −1 positions of the consensus donor splice site (Figure 7B and C). This indicates that the splicing events predominantly regulated by PRMT5 alone, or simultaneously by PRMT5 or PRMT4, are enriched in weak splice sites that deviate from the consensus sequence. Therefore, PRMT5 and PRMT4 are likely to regulate pre-mRNA splicing, at least in part, by contributing to stabilizing weak RNA-RNA interactions between donor splice sites that deviate from the consensus sequence, and the sequence present in the U1 SnRNA that is complementary to the consensus donor splice-site sequence. On the other hand, no deviation from the consensus sequence was observed for the acceptor splice site (data not shown), or for the donor splice site of the events affected only in *prmt4* mutants (Figure 7D). This observation suggests that PRMT4 may also contribute to the regulation of a subset of pre-mRNA splicing events acting, at least in part, by a different mechanism than PRMT5.

## Conclusions

Our comparative analysis of the physiological and molecular alterations present in *Arabidopsis prmt5* and *prmt4a;4b* mutants clearly shows that, in plants, the type II arginine methyltransferase PRMT5 and the type I methyltransferases PRMT4a and 4b most often co-regulate the same biological processes, affecting them in a similar manner. At the same time, we also found that some physiological processes are specifically affected by PRMT5 but not by PRMT4s. The transcriptome analysis conducted with RNA-seq revealed that the co-regulated nature of most of the physiological processes evaluated was more likely the result of overlapping roles of PRMT5 and PRMT4 in the regulation of transcription and pre-mRNA splicing. Thus, our findings suggest that the general idea that PRMT5 and PRMT4 play antagonist roles in the regulation of gene expression, may probably be a biased conclusion based on the analysis of few genes. Our study, besides being the first genome wide analysis of the effects of PRMT4 on gene expression and pre-mRNA splicing, strongly suggest that PRMT5 and PRMT4 have mostly similar molecular functions regulating gene expression at the transcriptional and post-transcriptional level.

## Methods

### Plant material

All of the Arabidopsis lines used in this study were in the Columbia ecotype. The *prmt5* mutant used was *prmt5-5* [25]. *prmt4a* (SALK_033423) and *prmt4b* (SALK_097442) mutants were obtained from the Arabidopsis Biological Research Center (ABRC) T-DNA insertion collections. The *prmt4a;4b* double mutant was obtained by crossing the simple mutants. The photoreceptor mutants used in this study were *phyB-9* and *cry1-b104;cry2-1*.

### Growth conditions

For flowering time experiments, the plants were grown on soil at 22°C under long days (LD; 16-h light/8-h dark cycles; 80 μmol.m^−2^.s^−1^ of white light) or short day (SD; 8-h light/16-h dark cycles; 140 μmol.m^−2^.s^−1^ of white light) depending on the experiment.

### Physiological measurements

Flowering time was estimated by counting the number of rosette leaves at the time of bolting. This experiment was replicated in four occasions with 15 plants of each genotype in each experiment. For leaf movement analysis, plants were grown under 16-h light/8-h dark cycles and transferred to continuous 20 μmol.m^−2^.s^−1^ white fluorescent light at 22°C, and the position of the first pair of leaves was recorded every 2 hours for 6 days using digital cameras and leaf angle was determined using ImageJ software [40]. Period estimates were calculated with Brass 3.0 software (Biological Rhythms Analysis Software System, available from http://www.amillar.org) and analyzed with the FFT-NLLS suite of programs [41]. An ANOVA followed by Tukey’s Multiple Comparison Test was used for comparisons among genotypes. For hypocotyl length measurements seedlings were grown on 0.8% agar under complete darkness, continuous red (0,01 to 100 μmol.m^−2^.s^−1^) and continuous blue light (0,1 to 10 μmol.m^−2^.s^−1^), and the final length of the hypocotyls was measured after 4 d. Light effects on hypocotyl elongation were calculated normalizing hypocotyl length under each light regime relative to hypocotyl length of the same genotype under constant dark conditions; N = 6 replicates of 10 seedlings each. An ANOVA followed by Tukey’s Multiple Comparison Test was used for comparisons among genotypes and irradiances. For salt stress assays seeds were germinated on MS agar medium, for the root growth assay 4 day old seedlings were transferred to MS agar containing 0 or 100 mM NaCl and then the seedlings were grown vertically for 10 days. Root growth was measured relative to that of plants kept under control conditions; more than 30 roots were measured for each data point. For the NaCl tolerance assay, 4 days old seedlings were transferred from the germination medium to MS agar containing 0 or 160 mM NaCl, and the survival rate was determined 30 days after the seedlings were transferred to the treatment medium.

### Growth conditions and protocol used for cDNA library preparation and high-throughput sequencing

Seeds were sown onto Murashige and Skoog medium containing 0.8% agarose, stratified for 4 d in the dark at 4°C, and then grown at 22°C in continuous light. Whole plants were harvested after 10 days, and total RNA was extracted with RNeasy Plant Mini Kit (QIAGEN) following the manufacturer’s protocols. To estimate the concentration and quality of samples, NanoDrop 2000c (Thermo Scientific) and the Agilent 2100 Bioanalyzer (Agilent Technologies) with the Agilent RNA 6000 Nano Kit were used, respectively. Libraries were prepared following the TruSeq RNA Sample Preparation Guide (Illumina). Briefly, 3 μg of total RNA was polyA-purified and fragmented, first-strand cDNA synthesized by reverse transcriptase (SuperScript III; Invitrogen) using random hexamers. This was followed by RNA degradation and second-strand cDNA synthesis. End repair process and addition of a single A nucleotide to the 3′ ends allowed ligation of multiple indexing adapters. Then, an enrichment step of 12 cycles of PCR was performed. Library validation included size and purity assessment with the Agilent 2100 Bioanalyzer and the Agilent DNA1000 kit (Agilent Technologies). Samples were pooled to create 12 multiplexed DNA libraries, which were pair-end sequenced with an Illumina HiSeq 1500 at INDEAR Argentina, providing 100-bp single-end reads. Three replicates for each genotype were sequenced. Sequencing data have been uploaded to the Gene Expression Omnibus database and hare available under accession number GSE62024.

### Processing of RNA sequencing reads

Sequence reads were mapped to Arabidopsis thaliana TAIR10 [42] genome using TopHat v2.0.9 [43] with default parameters, except of maximum intron length set at 5,000. Count tables for different feature levels were obtained from bam files using custom R scripts and considering TAIR10 transcriptome.

### Differential gene expression analysis

Before differential expression analysis, we decided to discard genes with fewer than 10 reads on average per condition. Differential gene expression was estimated using the edgeR package version 3.4.2 [44], and resulting P values were adjusted using a false discovery rate (FDR) criterion [45]. Genes with FDR values lower than 0.10 and absolute log-two fold change greater than 0.58 were deemed differentially expressed. Overlapping analysis were performed using Venny [46].

### Differential alternative splicing

For the analysis of alternative splicing, the transcriptome was partitioned into subgenic joint features called “bins,” as proposed on DEXseq [47]. Because of our special interest in new intron retention events not only exons but also introns were considered in our analysis. The transcriptome was partitioned into 281,321 bins; 152,631 corresponding exclusively to exonic regions, 120,717 to intronic regions, and 7,973 to DNA regions directly involved in alternatively spliced isoforms. We labeled these three kinds of bins as exon-bin, intron-bin, or AS-bins, respectively. In addition ASbins were further classified as exon skipping (ES), 5′ or 3′ alternative (5′alt, 3′alt), IR, or multiple (those including three or more different AS events in the same subgenic region) bins. For our analysis we discarded bins from monoexonic genes and with mean count values lower than 5 reads per condition. To provide a comprehensive summary of the calculated subgenic features, separate tables were produced for introns and AS-bins. We used edgeR exact test for the identification of differential use of bins corresponding to AS events or introns, and FDR-corrected P values. We also computed read densities to have a relationship between the bin and its corresponding gene. A Splicing Index was calculated as bin read density/gene read density, and the Splicing Index Ratio was calculated as Splicing Index in mutants/Splicing Index in wild-type plants. Only genes with read densities greater than 0.05 in all genotypes and Splicing Indexes greater than 0.05 in at least one genotype were used for the analysis. AS events as well as all introns with an absolute Log_2_ Fold Change (bin read density in the mutant/bin read density in wild-type) value greater than 0.58, with FDR values lower than 0.15, and an absolute Log_2_ Splicing Index Ratio (Splicing Index in the mutant/Splicing Index in WT) greater than 0.58 were deemed differentially spliced. Overlapping analysis were performed using Venny [46]. The custom R scripts used here are available upon request.

### Functional category enrichment analysis

Functional categories associated with specific groups of genes were identified using the BioMaps tool from the virtual plant software (http://virtualplant.bio.nyu.edu/cgi-bin/vpweb). This tool allowed us to determine which functional categories were statistically over represented in particular lists of genes compared to the entire genome [48].We analyzed fifteen functional categories of our interest, and for each one we determined the genes in common with our data sets, finally calculating a representation factor and the probability of finding an overlap simply by chance. The representation factor is the number of overlapping genes divided by the expected number of overlapping genes drawn from two independent groups. A representation factor > 1 indicates more overlap than expected by chance for two independent groups of genes or events, a representation factor < 1 indicates less overlap than expected. The probability of each overlapping was determined using the hypergeometric probability formula.

### Analysis of splice-site sequences

To evaluate possible changes in the splice-site sequences of the most significantly affected splicing events in the *prmt* mutants, we obtained the donor and acceptor splice site sequences of all the intron retention events that were changed at least two fold (splicing index ratio ≥1 or ≤1 and FDR ≤0.1) in *prmt* mutants compared to wild type plants, and compared them to the consensus splice-site sequences of the 119,072 GT_AG_U2 introns present in *Arabidopsis.* The frequency of each nucleotide for each position was obtained using the Unipro UGENE software (Additional file 5) [49], and were represented using the R package Seqlogo [50]. The over- or under-representation of a particular nucleotide relative to its genome-wide frequency was determined and a p-value for the analysis was obtained using the hypergeometric test.

### PCR alternative splicing assessment

PCR amplification was performed using 1.5 U of Taq polymerase (Invitrogen). Primers used for amplification are detailed in Additional file 6. RT–PCR products were electrophoresed and detected by SYBR Green 2% (for AT3G17100 and AT5G63460) or Ethidium Bromide 2% (for AT2G46790).

## Availability of supporting data

The data sets supporting the results of this article are available in the Gene Expression Omnibus (GEO) repository, http://www.ncbi.nlm.nih.gov/geo/query/acc.cgi?acc=GSE62024.

## Additional files

Additional file 1:
**RNAseq Expression Data.** Differentially expressed genes in *prmt5* and *prmt4a;4b* mutants, relative to the wild-type. The FDR and Log_2_ FC are displayed. Additional file 2:
**Impact of PRMT5 and PRMT4s on genome wide AS events distribution.** Percentage composition of AS events according to their category: 3′ Alt: 3′ alternative splicing site, 5′ Alt: 5′ alternative splicing site, IR: Intron Retention, ES: Exon Skipping. Here are displayed the distribution in categories of all AS altered events in WT plants and both *prmt5* and *prmt4a;4b* mutants. Additional file 3:
**RNAseq AS Data.** Differentially affected alternative splicing events in *prmt5* and *prmt4a;4b* mutants, relative to the wild-type. The alternative BIN, event category, gene coordinates, read densities and splicing ratios are displayed. Additional file 4:
**RNAseq Constitutive Splicing Data.** Differentially affected constitutive splicing events in *prmt5* and *prmt4a;4b* mutants, relative to the wild-type. The alternative BIN, event category, gene coordinates, read densities and splicing ratios are displayed. Additional file 5:
**Bioinformatic analysis of donor splice-site sequences.** For each data set analyzed are displayed the frequency of each nucleotide for each position, the representation factor and the P value. Additional file 6:
**Splicing events validation primers.** List of all primers used for validation of the alternative splicing events previously described.

## Abbreviations

aDMA: Asymmetric dimethylarginines
sDMA: Symmetric dimethylarginines
AS: Alternative splicing
*prmt4a*;4b: Double mutant affected in the *PRMT4a* and *PRMT4b* genes
