# Supplementary figures and images for: Genome wide comparative analysis of the effects of PRMT5 and PRMT4/CARM1 arginine methyltransferases on the Arabidopsis thaliana transcriptome
Source: BMC Genomics. 2015 Mar 17;16(1):192. doi: 10.1186/s12864-015-1399-2 (PMC4381356; doi:10.1186/s12864-015-1399-2)

Total AS Events in WT  
(5362)

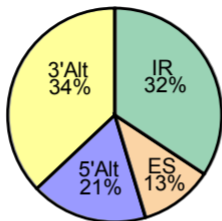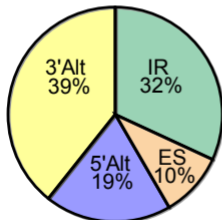

AS Events Affected  
in *prmt5*  
(1137)

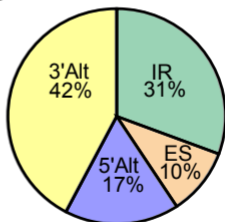

AS Events Affected  
in *prmt4a;4b*  
(1290)

Supplement: Additional file 2: — Impact of PRMT5 and PRMT4s on genome wide AS events distribution. Percentage composition of AS events according to their category: 3′ Alt: 3′ alternative splicing site, 5′ Alt: 5′ alternative splicing site, IR: Intron Retention, ES: Exon Skipping. Here are displayed the distribution in categories of all AS altered events in WT plants and both prmt5 and prmt4a;4b mutants. [file 12864_2015_1399_MOESM2_ESM.pdf]
